# Temporary cessation of ibrutinib results in reduced grade 3‐4 infections and durable remissions—Interim analysis of an on‐off‐repeat Phase 1b/2 study in patients with chronic lymphocytic leukemia

**DOI:** 10.1002/jha2.261

**Published:** 2021-07-14

**Authors:** Jeanette Lundin, Tom A. Mulder, Magdalena Kättström, Tove Wästerlid, Anders Uddevik, Håkan Mellstedt, Kia Heimersson, Lotta Hansson, Marzia Palma, Anders Österborg

**Affiliations:** ^1^ Department of Oncology‐Pathology Karolinska Institutet Stockholm Sweden; ^2^ Lymphoma Unit, Department of Hematology Karolinska University Hospital Solna Stockholm Sweden; ^3^ Department of Medicine, Section of Hematology Örebro University Hospital Örebro Sweden; ^4^ Division of Clinical Epidemiology, Department of Medicine Solna Karolinska Institutet Stockholm Sweden; ^5^ Department of Medicine, Section of Hematology Gävle Hospital Gävle Sweden

## Abstract

Ibrutinib is used continuously in CLL. This phase 1b/2 study interim analysis explored on‐off‐repeat dosing to reduce toxicity. After 12 months, 16/22 patients (73%) remained in first off‐phase irrespective if initial CR/PR or *TP53* aberration. Grade 3‐4 infections were reduced from 55% to 5% during a similarly long off‐phase (*P* < .01). Treg and exhausted T‐cells increased (*P* = .01). Six patients restarted ibrutinib at early progression and remain drug‐sensitive. Our interim analysis shows a durable off‐phase in most patients, with reduced infections and cost‐saving potential. If toxicity‐driven permanent cessation of ibrutinib will be affected will be explored in the extended study.

## 1 INTRODUCTION

Most patients with chronic lymphocytic leukemia (CLL) relapse after first‐line chemoimmunotherapy and treatment with ibrutinib, an inhibitor of Bruton's tyrosine kinase (BTK), has improved prognosis of relapsed/refractory (R/R) patients in trials [1, 2] and real‐world reports. [3] However, long‐term continuous usage is burdened by side effects such as hematomas, atrial fibrillation, hair, skin, and nail changes and high annual costs for the society. A real‐world analysis on strictly consecutive patients receiving continuous ibrutinib showed a median time to permanent cessation of ibrutinib of only 18 months, mainly due to toxicity. [4] The risk of opportunistic and other infections during ibrutinib therapy has been debated. [5] Observations in patients who stopped ibrutinib treatment permanently due to side effects suggest that clinical responses may be long‐lasting. [6] Thus, we raised the question whether ibrutinib could safely be interrupted in responding CLL patients and restated at early signs of progression, reducing side effects and possibly the risk of permanent drug cessation. Maintaining ibrutinib tolerability may be crucial to delay the need for alternative therapy. Ibrutinib may not be cost‐effective with the current standard dosing [7] and repetitive on‐off dosing may also have significant impact on total drug costs.

### 1.1 Study design

We report here early interim results from a Phase 1b/2 multicenter, prospective, single‐arm, investigator‐initiated study of CLL patients who were in remission following continuous ibrutinib therapy, designed to investigate the safety and efficacy of repeated response‐guided treatment interruptions. The primary study endpoints were safety, that is, adverse events (AEs) and relationship of AEs to intermittent and repeated ibrutinib dosing, as well as the time to need of alternative therapy other than ibrutinib. Secondary endpoints were overall response and time to response, progression free survival, time from stop until restart of each ibrutinib cycle, number of ibrutinib cycles, cumulative dose of ibrutinib, overall survival and changes in biological and immunological variables at stop and start of each cycle. The study was approved by the Ethics authority and Medical Product Agency of Sweden. All patients provided written informed consent.

A preplanned early interim analysis was scheduled after 20 included patients; the number was based on our previous experience on explorative studies to provide an indicator that no safety signals emerged and that outcome measures showed a positive trend. [8, 9, 10] Eligibility criteria are: CLL with treatment indication according to the iwCLL criteria before start of continuous ibrutinib therapy in the R/R setting or first or later line in case of *TP53* aberration; having received ≥6 months of ibrutinib therapy; stable clinical partial remission (PR) according to iwCLL criteria or better; aged ≥18 years; ECOG ≤2; neutrophil count ≥0.5 × 10^9^/L; platelet count ≥30 × 10^9^/L; serum creatinine < 177 μmol/L.

Computed tomography (CT) and bone marrow examinations are performed at study inclusion. Ibrutinib is stopped (study entry, first off‐phase) and patients are followed closely. At early signs of progressive disease (PD) defined as new enlarged lymph node ≥2 cm and/or >50% increase of existing lymph nodes and/or >50% increased lymphocytosis and/or lymphocytosis of >30 × 10^9^/L, ibrutinib is restarted (on‐phase). Treatment will then continue until a new stable PR is achieved and then stopped again (second off‐phase). Such cycles are planned to be repeated until nontolerability or nonresponsiveness to ibrutinib forced permanent discontinuation.

Lab tests and telephone contact are mandatory every second week during the first 2 months to rule out rebound phenomena following drug cessation. Physical examination is performed monthly during the first 6 months of every on‐ and off‐phase and every other month thereafter. Blood counts with differential are performed every second week during the first two months of every off‐phase and monthly thereafter, and monthly during every on‐phase. In case of early signs of PD, a more comprehensive evaluation, which included a CT and bone marrow biopsy is performed before ibrutinib is restarted. With every subsequent on‐off cycle the same comprehensive evaluation is performed.

Immunomonitoring is performed during the first off‐phase (baseline, 2 and 4 weeks, 3, 6, and 12 months). Materials and methods are included as supplement.

## 2 RESULTS AND DISCUSSION

We report here the early preplanned interim analysis of the Phase 1b/2 study. A total of 22 patients were enrolled during 20 months at three sites. Four patients were included in an early run‐in safety period and had to complete 6 months of the first off‐phase before additional patients could be included in the study. Patients characteristics are shown in Table 1. Median age was 73.5 years (range 52‐86), nine patients (41%) had *TP53*‐mutation and/or 17p deletion, and eight patients (36%) received ibrutinib as first‐line treatment.

**TABLE 1:** Patient characteristics at baseline and side effects during 12 months before and during 1st off‐phase

Patient characteristics at study inclusion (*n* = 22)		
Gender		
Male, *n* (%)	16 (73)	
Female, *n* (%)	6 (27)	
Age (years), median (range)	73.5 (52‐85)	
Performance status (ECOG)		
0, *n* (%)	16 (73)	
1, *n* (%)	6 (27)	
Fluorescence in situ hybridization (FISH) and/or array		
*TP53*‐mut/del(17p), *n* (%)	9 (41)	
Del(13q), *n* (%)	12 (55)	
Del(11q), *n* (%)	3 (14)	
Trisomy 12, *n* (%)	1 (5)	
Normal, *n* (%)	3 (14)	
Not done, *n* (%)	2 (9)	
Time (months) since CLL diagnosis at study entry, median (range)	90 (33‐206)	
Number of previous treatments, median (range)	2 (1‐6)	
Ibrutinib treatment:		
First line, *n* (%)	8 (36)	
Second or later line, *n* (%)	14 (64)	
Duration (months) of prior ibrutinib treatment at study entry, median (range)	26 (7‐77)	
Response at study entry		
PR, *n* (%)	11 (50)	
CR, *n* (%)	11 (50)	
Follow‐up on study (months), median (range)	13 (9‐32)	
**Side effects observed during 12 months prior to study inclusion and during the first off‐phase**		
Grade 1‐2 hematoma/bleeding		
Before study inclusion, *n* (%)	13 (59)	*P* = .0015*
During the first off‐phase, *n* (%)	1 (5)	
Grade 1‐2 Fragile nails and/or skin (fingertip) eruptions		
Before study inclusion, *n* (%)	10 (45)	*P* = .0044*
During the first off‐phase, *n* (%)	0 (0)	
Grade ≤2 infections		
Before study inclusion, *n* (%)	12 (55)	NS
During the first off‐phase, *n* (%)	7 (32)	
Grade ≥3 infections		
Before study inclusion, *n* (%)	12** (55)	*P* = .0055*
During the first off‐phase, *n* (%)	1 (5)	
Grade 1‐2 arthralgias		
Before study inclusion, *n* (%)	2 (9)	NS
During the first off‐phase, *n*	1 (5)	
Atrial fibrillation		
Before study inclusion, *n* (%)	2 (9)	NS
During the first off‐phase, *n* (%)	(0)	
Hypertension		
Before study inclusion, *n* (%)	0	NS
During the first off‐phase, *n* (%)	0	

* *P* values refer to McNemar test.

** Sepsis or neutropenic fever, *n* = 6, pneumonia, *n* = 6.

**FIGURE 1:**
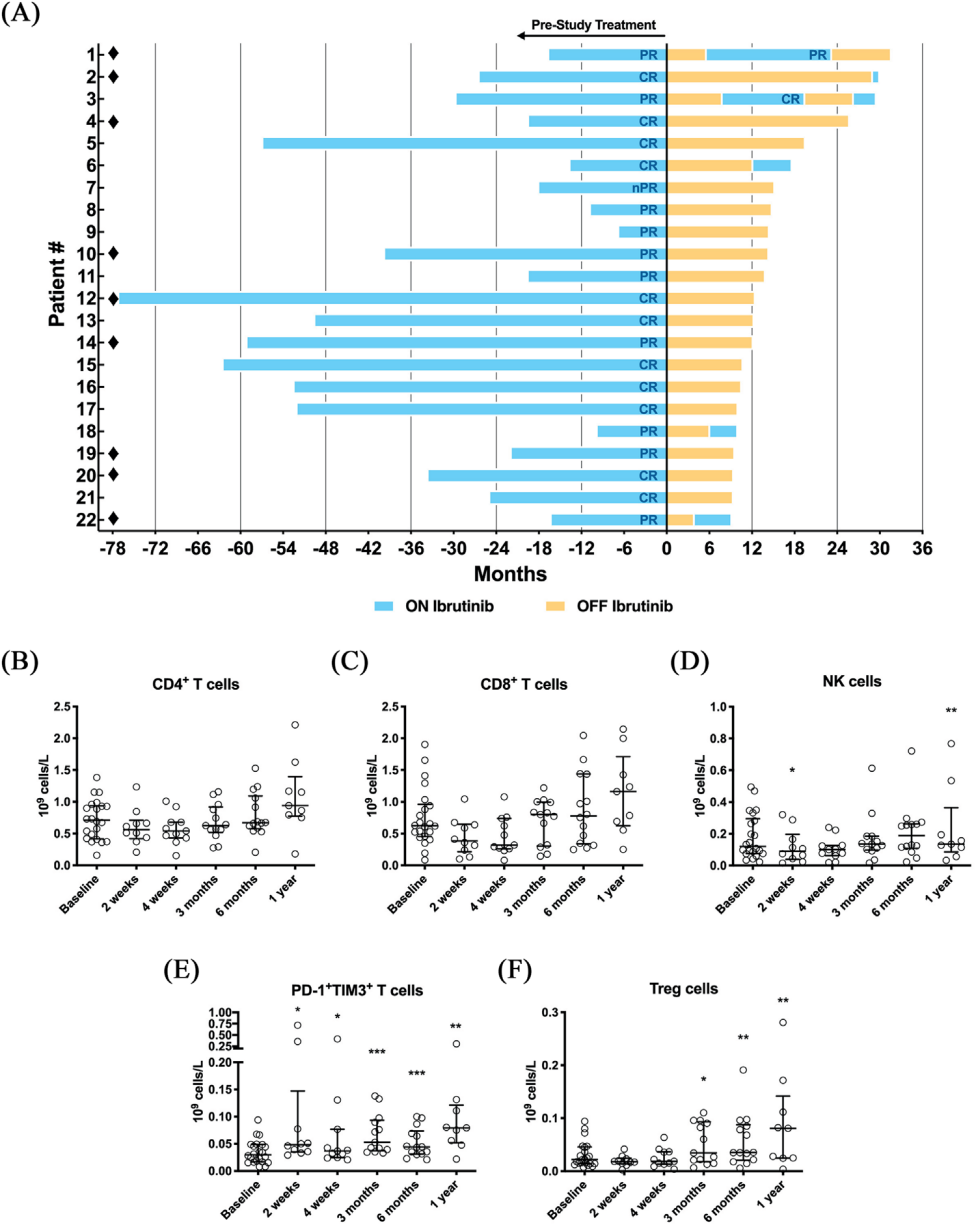
Response‐guided on‐off dosing in ibrutinib‐treated CLL patients and immunomonitoring during the first year of the initial off‐phase. (A) Duration of ibrutinib treatment prior to study enrollment (Months = 0) and duration of the respective response‐guided interruptions (off; in orange) and resumptions (on; in blue). Quality of response (CR or PR) at the time of ibrutinib cessation is indicated at the end of each on‐phase. Absolute numbers of (B) CD3^+^CD4^+^, (C) CD3^+^CD8^+^, (D) CD3^−^CD56^+^, (E) CD3^+^PD‐1^+^TIM3^+^, and (F) CD4^+^CCR4^+^CD25^+^CD127^low^ lymphocytes, respectively, in the peripheral blood during the first year of the initial off‐phase as measured by flow cytometry. Panels B to F show data from 14 patients at ibrutinib cessation (study baseline) and the indicated period thereafter. Stars indicate a statistically significant difference between the respective timepoints and baseline as established by Wilcoxon signed‐rank test. *P ≤ .05; **P ≤ .01; ***P ≤ .001; CR: complete remission; PR: partial remission; MRD; minimal residual disease; PD‐1: programmed cell death protein 1; TIM3: T cell immunoglobulin and mucin domain‐containing protein 3; Treg: regulatory T cell; ♦: patients with 17 p deletion and/or *TP53* mutation

Of the 22 patients, 11 (50%) entered the study with a PR and 11 with CR, which was minimal residual disease (MRD)‐positive in all cases (Figure 1A). Rapid progressive disease (rebound) or other safety signals were not observed. Sixteen patients (73%) remained in their first off‐phase without early signs of PD at the 12 months follow‐up (Figure 1A). Median time to progression has not been reached. There was tendency toward a longer off‐phase among patients with clinical CR, even though many patients with PR at inclusion also remained off drug at the 12‐month follow‐up (Figure 1A). Cytogenetic status was not related to length of first off‐phase, but longer follow‐up and extended number of patients will be required. Six patients (27%) showed early signs of progression (for criteria see “Study design” above) and restarted ibrutinib after a median of 7 months (range 4‐29); all responded with shrinking lymph nodes and/or improving blood counts. In one patient, the second response was deeper (MRD‐positive CR) than achieved prior to study inclusion (PR). However, this patient experienced increasing lymphocytosis after 7 months in the second off‐phase and restarted ibrutinib and has achieved a new PR.

We thereafter compared side effects observed during 12 months before study inclusion and during the first off‐phase, that is, a similarly long time period off‐drug (Table 1). Fragile nails and/or skin (fingertip) eruptions were present in 10 patients prior to study inclusion; not unexpectedly, all of them had normalized nails and fingertips during the first off‐phase (*P* = .0044, McNemar's test). Similarly, hematomas or bleeding disappeared in 12/13 patients during the off‐phase (*P* = .0015). Two patients had developed atrial fibrillation and arthralgias, respectively, during the last 12 months before study entry. No new cases were observed during the off‐phase. Notably, the frequency of grade ≥3 infections was significantly lower (*P* = .0055) during the first off‐phase (5%) than in the preceding 12 months on ibrutinib (55%). There was no significant difference in grade ≤2 infections (55% before vs 32% after drug cessation). The reason for the reduced risk of severe infections is not clear since grade 4 neutropenia and hypogammaglobulinemia did not differ between the two time periods. We assume that the lower incidence of infections was ascribable to the patients being off the drug. Several studies have shown improved humoral immunity (especially IgA) during ibrutinib treatment. [11] Whether humoral immunity may improve following cessation of ibrutinib will be object of future investigations including vaccine trials.

We previously showed that most peripheral blood T‐cell subsets decline during ibrutinib treatment, which correlates with a decreasing disease burden. [12] No significant changes in the numbers of CD4^+^ and CD8^+^ T‐cell subsets were observed following ibrutinib cessation (Figure 1B and C). NK cells (CD3^−^CD56^+^) decreased during the first 2 weeks (*P* = .04) but then increased (*P* = .008 at 12 months) (Figure 1D). Exhausted T cells (CD3^+^PD‐1^+^TIM3^+^) (*P* = .01 after 2 weeks) and regulatory T cells (*P* = .01 after 3 months) gradually increased during the first off‐phase (Figure 1E, F). Such immune defects are likely to reflect the tumor burden. [13, 14, 15]

There are several limitations in our study. First, the data presented here is an early interim analysis, that is, on limited number of patients and need to be confirmed in more patients (ongoing). Since cessation of a BTK inhibitor in responding patients is outside the current treatment recommendation, we decided to perform an early interim analysis to check safety signals and trends for efficacy to decide whether continuation of the study is justified. Our early data suggest that severe infections may be reduced and that the first drug‐free period exceeded one year in most patients, which is considered as clinically meaningful. Furthermore, all re‐treated patients remain drug‐sensitive. Second, the study design may not be optimal; an alternative design would be to use a predefined length of ibrutinib therapy or a predefined depth of remission before drug cessation. However, our early data suggest that neither length of therapy nor degree of remission may be critical for the length of first off‐phase. Third, even though the cost‐saving potential is obvious in a short term perspective, longer follow‐up (including more patients and repeated on‐off cycles) will be required to determine all aspects of our dosing strategy—only a randomized comparison can provide the final answer on whether ibrutinib and other BTKi should be continued until PD or if they can safely be halted. Since half of patients treated with ibrutinib continuously in a real‐world setting had stopped ibrutinib permanently already at 18 months [4] we suggest that an alternative dosing strategy shall be developed to minimize permanent withdrawals, thereby possibly delay the time to need of alternative therapy.

## AUTHOR CONTRIBUTIONS

JL and AÖ designed the study, treated the patients, analyzed the data, and wrote the manuscript. TAM designed and performed laboratory experiments, analyzed the data, performed statistical analysis, and wrote the manuscript. MK, LH, and AU treated the patients and approved the manuscript. HM analyzed the immunological data and approved the manuscript. TW analyzed the clinical data and approved the manuscript. KH designed and performed laboratory experiments, analyzed the data, and approved the manuscript. MP designed laboratory experiments and wrote the manuscript.

## CONFLICT OF INTEREST

All authors declare no conflict of interest.

## Supporting information

Supporting Information

## References

[1] Byrd JC , Furman RR , Coutre SE , Flinn IW , Burger JA , Blum KA , et al. Targeting BTK with ibrutinib in relapsed chronic lymphocytic leukemia. N Engl J Med. 2013;369(1):32–42. 23782158 10.1056/NEJMoa1215637 PMC3772525

[2] O'Brien S , Jones JA , Coutre SE , Mato AR , Hillmen P , Tam C , et al. Ibrutinib for patients with relapsed or refractory chronic lymphocytic leukaemia with 17p deletion (RESONATE‐17): a phase 2, open‐label, multicentre study. Lancet Oncol. 2016;17(10):1409–18. 27637985 10.1016/S1470-2045(16)30212-1

[3] Winqvist M , Asklid A , Andersson PO , Karlsson K , Karlsson C , Lauri B , et al. Real‐world results of ibrutinib in patients with relapsed or refractory chronic lymphocytic leukemia: data from 95 consecutive patients treated in a compassionate use program. A study from the Swedish Chronic Lymphocytic Leukemia Group. Haematologica. 2016;101(12):1573–80. 27198718 10.3324/haematol.2016.144576 PMC5479603

[4] Andersson MM , Österborg S , Månsson‐Broberg A , Hansson A , Palma LM , Incidence of cardiovascular and bleeding events in chronic lymphocytic leukemia patients (CLL) treated with ibrutinib—a Swedish single‐center experience. Abstract XIX International Workshop on CLL (iwCLL), 2021 (virtual meeting).

[5] Ryan CE , Cheng MP , Issa NC , Brown JR , Davids MS . Pneumocystis jirovecii pneumonia and institutional prophylaxis practices in CLL patients treated with BTK inhibitors. Blood Adv. 2020;4(7):1458–63. 32282880 10.1182/bloodadvances.2020001678 PMC7160295

[6] Winqvist M , Andersson PO , Asklid A , Karlsson K , Karlsson C , Lauri B , et al. Long‐term real‐world results of ibrutinib therapy in patients with relapsed or refractory chronic lymphocytic leukemia: 30‐month follow up of the Swedish compassionate use cohort. Haematologica. 2019;104(5):e208–e10. 30514799 10.3324/haematol.2018.198820 PMC6518914

[7] Patel KK , Isufi I , Kothari S , Davidoff AJ , Gross CP , Huntington SF . Cost‐effectiveness of first‐line vs third‐line ibrutinib in patients with untreated chronic lymphocytic leukemia. Blood. 2020;136(17):1946‐55. 32518952 10.1182/blood.2020004922

[8] Lundin J , Kimby E , Bjorkholm M , Broliden PA , Celsing F , Hjalmar V , et al. Phase II trial of subcutaneous anti‐CD52 monoclonal antibody alemtuzumab (Campath‐1H) as first‐line treatment for patients with B‐cell chronic lymphocytic leukemia (B‐CLL). Blood. 2002;100(3):768–73. 12130484 10.1182/blood-2002-01-0159

[9] Winqvist M , Mozaffari F , Palma M , Eketorp Sylvan S , Hansson L , Mellstedt H , et al. Phase I‐II study of lenalidomide and alemtuzumab in refractory chronic lymphocytic leukemia (CLL): effects on T cells and immune checkpoints. Cancer Immunol Immunother. 2017;66(1):91–102. 27815572 10.1007/s00262-016-1922-6 PMC5222940

[10] Winqvist M , Palma M , Heimersson K , Mellstedt H , Osterborg A , Lundin J . Dual targeting of Bruton tyrosine kinase and CD52 induces minimal residual disease‐negativity in the bone marrow of poor‐prognosis chronic lymphocytic leukaemia patients but is associated with opportunistic infections—results from a phase I study. Br J Haematol. 2018;182(4):590–4. 28677818 10.1111/bjh.14836

[11] Sun C , Tian X , Lee YS , Gunti S , Lipsky A , Herman SE , et al. Partial reconstitution of humoral immunity and fewer infections in patients with chronic lymphocytic leukemia treated with ibrutinib. Blood. 2015;126(19):2213–9. 26337493 10.1182/blood-2015-04-639203 PMC4635117

[12] Palma M , Heimersson K , Mulder TA , Nasman‐Glaser B , Osterborg A , Mellstedt H . Reduction of tumor burden rather than off‐target effects drives changes in T‐cell number and profile during prolonged ibrutinib treatment in relapsed or refractory chronic lymphocytic leukemia patients. Blood. 2018;132.29866817

[13] Palma M , Gentilcore G , Heimersson K , Mozaffari F , Nasman‐Glaser B , Young E , et al. T cells in chronic lymphocytic leukemia display dysregulated expression of immune checkpoints and activation markers. Haematologica. 2017;102(3):562–72. 27927767 10.3324/haematol.2016.151100 PMC5394965

[14] Riches JC , Davies JK , McClanahan F , Fatah R , Iqbal S , Agrawal S , et al. T cells from CLL patients exhibit features of T‐cell exhaustion but retain capacity for cytokine production. Blood. 2013;121(9):1612–21. 23247726 10.1182/blood-2012-09-457531 PMC3587324

[15] Taghiloo S , Allahmoradi E , Tehrani M , Hossein‐Nataj H , Shekarriz R , Janbabaei G , et al. Frequency and functional characterization of exhausted CD8(+) T cells in chronic lymphocytic leukemia. Eur J Haematol. 2017;98(6):622–31. 28306177 10.1111/ejh.12880

