# Supplementary material for: Temporary cessation of ibrutinib results in reduced grade 3‐4 infections and durable remissions—Interim analysis of an on‐off‐repeat Phase 1b/2 study in patients with chronic lymphocytic leukemia
Source: EJHaem. 2021 Jul 14;2(3):525–9. doi: 10.1002/jha2.261 (PMC9176042; doi:10.1002/jha2.261)
Supplement: Supplementary file 1 — Supporting Information [file JHA2-2-525-s001.docx]

**Supplemental data**

**Materials and methods**

**Flow cytometric characterization of lymphocyte subsets**

Fresh whole blood cells were stained with in 2 different monoclonal antibody panels. The first panel contained CD3-PE-Cy7 (HIT3a), CD4-PerCP (RPA-T4), CD8-APC (SK1), CD16-PE (3G8), CD19-AF488 (HIB19), CD45-AF700 (HI30) and CD56-PE (HCD56) (BioLegend, San Diego, CA, USA). The second panel contained CD3-AF700 (UCHT1), CD16-PE-Cy7 (3G8), CD19-PerCp (HIB19), CD56-PE (HCD56) and PD-1 APC (EH12.2H7) (BioLegend), and CD366-BB515 (7D3) (BD Biosciences, San Diego, CA, USA). Then, these suspensions were subjected to red blood cell lysis, washed once in Cell Staining Buffer (CSB) (BioLegend) and resuspended in CSB before data acquisition on a FACSCanto II flow cytometer (BD Biosciences).

Peripheral blood mononuclear cells (PBMC) were isolated from whole heparinized blood by density gradient centrifugation using a Ficoll-Hypaque gradient (GE Healthcare, Uppsala, Sweden) and washed twice in Dulbecco’s Phosphate-Buffered Saline (DPBS) (Gibco, Life Technologies, Carlsbad, CA, USA). Cells were stained freshly with the following monoclonal antibodies: CCR4-PE (1G1) and CD127-PE-Cy7 (HIL-7R-M21) (BD Biosciences), and CD4-AF700 (OKT4), CD25-APC (BC96), CD45RO-FITC (UCHL1) HLA-DR-PerCP (L243) (BioLegend). Cells were subsequently washed in CSB before data acquisition on a FACSCanto II flow cytometer.

Analysis was performed using FACSDiva software version 6.1.3 (BD Biosciences).
